# Intraperitoneal but Not Intravenous Cryopreserved Mesenchymal Stromal Cells Home to the Inflamed Colon and Ameliorate Experimental Colitis

**DOI:** 10.1371/journal.pone.0033360

**Published:** 2012-03-14

**Authors:** Morgana T. L. Castelo-Branco, Igor D. P. Soares, Daiana V. Lopes, Fernanda Buongusto, Cesonia A. Martinusso, Alyson do Rosario, Sergio A. L. Souza, Bianca Gutfilen, Lea Mirian B. Fonseca, Celeste Elia, Kalil Madi, Alberto Schanaider, Maria Isabel D. Rossi, Heitor S. P. Souza

**Affiliations:** 1 Laboratório de Imunologia Celular, Instituto de Ciências Biomédicas, Universidade Federal do Rio de Janeiro, Rio de Janeiro, Brazil; 2 Laboratório Multidisciplinar de Pesquisa, Departamento de Clínica Médica, Universidade Federal do Rio de Janeiro, Rio de Janeiro, Brazil; 3 Laboratório de Imunohematologia, Departamento de Clínica Médica, Universidade Federal do Rio de Janeiro, Rio de Janeiro, Brazil; 4 Serviço de Medicina Nuclear, Laboratório de Marcação de Células e Moléculas (LMCM), Departamento de Radiologia, Universidade Federal do Rio de Janeiro, Rio de Janeiro, Brazil; 5 Departamento de Patologia, Universidade Federal do Rio de Janeiro, Rio de Janeiro, Brazil; 6 Laboratório de Cirurgia Experimental, Departamento de Cirurgia, Universidade Federal do Rio de Janeiro, Rio de Janeiro, Brazil; Federal University of Rio de Janeiro, Brazil

## Abstract

**Background and Aims:**

Mesenchymal stromal cells (MSCs) were shown to have immunomodulatory activity and have been applied for treating immune-mediated disorders. We compared the homing and therapeutic action of cryopreserved subcutaneous adipose tissue (AT-MSCs) and bone marrow-derived mesenchymal stromal cells (BM-MSCs) in rats with trinitrobenzene sulfonic acid (TNBS)–induced colitis.

**Methods:**

After colonoscopic detection of inflammation AT-MSCs or BM-MSCs were injected intraperitoneally. Colonoscopic and histologic scores were obtained. Density of collagen fibres and apoptotic rates were evaluated. Cytokine levels were measured in supernatants of colon explants. For cell migration studies MSCs and skin fibroblasts were labelled with Tc-99m or CM-DiI and injected intraperitonealy or intravenously.

**Results:**

Intraperitoneal injection of AT-MSCs or BM-MSCs reduced the endoscopic and histopathologic severity of colitis, the collagen deposition, and the epithelial apoptosis. Levels of TNF-α and interleukin-1β decreased, while VEGF and TGF-β did not change following cell-therapy. Scintigraphy showed that MSCs migrated towards the inflamed colon and the uptake increased from 0.5 to 24 h. Tc-99m-MSCs injected intravenously distributed into various organs, but not the colon. Cm-DiI-positive MSCs were detected throughout the colon wall 72 h after inoculation, predominantly in the submucosa and muscular layer of inflamed areas.

**Conclusions:**

Intraperitoneally injected cryopreserved MSCs home to and engraft into the inflamed colon and ameliorate TNBS-colitis.

## Introduction

Inflammatory bowel disease (IBD) comprises a spectrum of chronic and relapsing diseases including Crohn's disease (CD) and ulcerative colitis. CD is characterized by a background of mucosal T-cell dysfunction, inflammatory cell infiltration, and abnormal cytokine production leading to uncontrolled and persistent intestinal transmural inflammation [1]. Although the aetiology of CD remains unknown, there is evidence indicating the existence of an abnormal immune response against the gut comensal microbiota [2]. However, despite all recent scientific advances in the study of IBD, available therapies for CD are still largely based on non-specific immunosuppressive agents, and continue to have limited efficacy with major concerns regarding safety issues [3]. Indeed, this illustrates the need for investigating new therapeutic alternatives to dampen local inflammation, both effectively modulating Th1-driven response and restoring the integrity of the mucosal barrier.

Mesenchymal stromal cells (MSCs) are adult somatic cells that reside in the stroma of solid organs and are considered as precursors of non-haematopoietic connective tissues. MSCs have been demonstrated to differentiate into cells of mesodermal lineage such as bone, cartilage, and fat [4]. Recently, MSC have been explored in regenerative medicine due not only to their differentiation potential but mostly to the capacity to improve the repair of damaged tissues by secretion of biological active molecules [5]. In addition MSCs are also capable of actions such as inhibiting T-cell proliferation [6], inducing T-cell apoptosis [7], and blocking dendritic cell maturation [8]. Because of their low immunogenicity profile MSCs can be successfully transplanted in diverse allogeneic and xenogeneic settings [9]. Recently, MSCs obtained from the subcutaneous adipose tissue (AT-MSCs), with basically the same immunomodulatory properties as bone marrow mesenchymal stromal cells (BM-MSCs), have risen as a new therapeutic perspective for cell-based therapies [10], [11].

Successful application of AT-MSCs has been reported recently in experimental colitis [12]–[14]. Nevertheless, in view of the different protocols utilized in those studies, the biological mechanisms underlying the beneficial effect and immunoregulatory activity of MSCs are yet to be determined. Therefore, here we investigated in vivo the potential therapeutic effect of non-myeloabaltive transplantation of cryopreserved allogeneic AT- and BM-MSCs in trinitrobenzene sulfonic acid (TNBS)-induced colitis, an established experimental model of CD [15]. Follow-up colonoscopic assessment of animals allowed us to confirm colitis induction and to grade inflammation in live animals. Furthermore, we also investigated recruitment to the intestinal mucosa of MSCs upon different routes of administration.

## Results

### Characterization of MSCs from Subcutaneous Adipose Tissue and Bone Marrow

**Figure 1:**
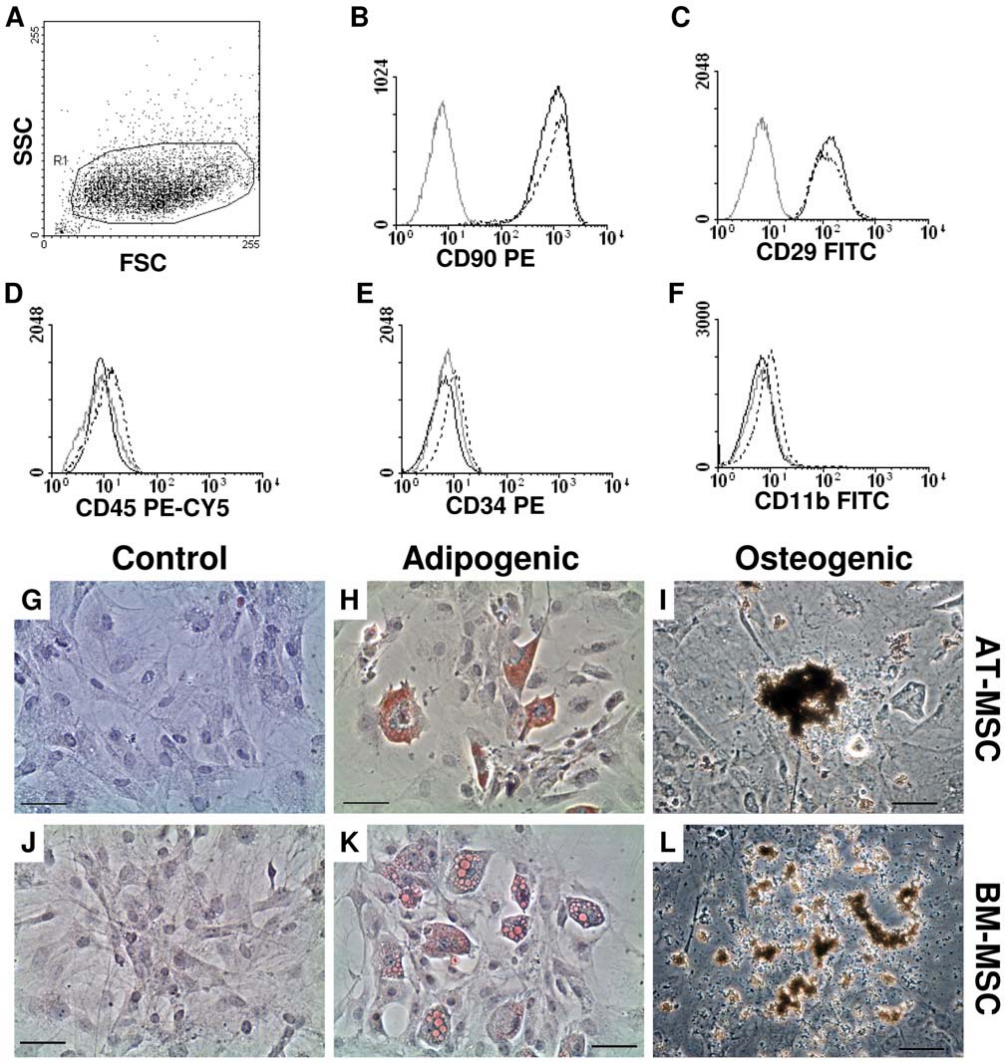
Biological characterization of mesenchymal stromal cells. Phenotypic analysis of MSCs was carried out by flow cytometry, which revealed that BM- and AT-MSCs expressed the cell markers CD90 (Thy-1) and CD29, but did not express lineage markers such as CD45, CD11b and CD34 (A–F). Solid black lines show AT-MSC, dotted black lines show BM-MSC, and gray lines are isotype control. Functionally, MSCs have the capacity to form different cell lineages. AT- (G–I) and BM- (J–K) MSCs were able to differentiate into adipocytes (H, K) and osteocytes (I, L). These cells were used in subsequent experiments. Oil Red O (G–H, J–K) and Von Kossa (I, L) staining. Length bars represent 50 µm.

AT- and BM-MSCs were flow-cytometrically characterized (after 3 passages). AT- and BM-MSCs were positive for CD90 and CD29, but negative for CD45, CD11b, and CD34 (Figure 1A–F), indicating that no haematopoietic cells remained in the MSCs suspension used in our experiments. Of note, the doubling time of AT- and BM-MSCs remained unchanged (approximately 36–40 hours) up to 7 passages, but their growth stopped at 14 passages.

### Multilineage Potential of AT- and BM-MSCs

To examine the ability to differentiate into multilineage cells, of AT- and BM-MSCs were cultured with the addition of lineage-specific induction factors. Adipogenic differentiation was confirmed by Oil Red-O staining (Figure 1H, K). Osteogenic differentiation was determined by extra cellular matrix calcification detected by Von Kossa staining (Figure 1I, L).

### Intraperitoneal Inoculation of MSCs Ameliorates TNBS-Induced Colitis

We first investigated the potential therapeutic effect of MSCs on the TNBS-induced colitis model, which displays a predominant Th1-mediated immune response characterized by mononuclear cells infiltration throughout the colon wall, similar to human CD [15], [16]. Animals exposed to TNBS developed an extensive and severe colitis, characterized by diarrhoea, and accompanied by a wasting syndrome with remarkable weight loss.

**Figure 2:**
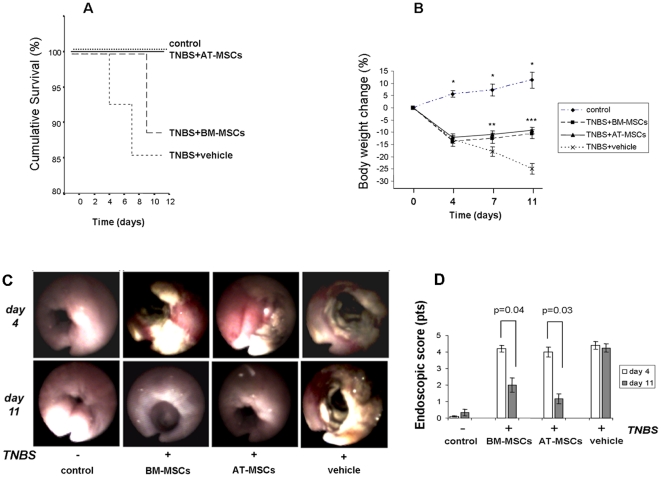
Effect of MSCs on the clinical and colonoscopic parameters of the colitis model. Survival did not differ significantly among experimental groups. Of the TNBS-colitic animals, 2 treated with vehicle died on days 4 and 7, and 1 treated with BM-MSCs died on day 9. Survival was analyzed by the Kaplan–Meier log-rank test (A). Following TNBS-induction, animals presented a progressive weight loss compared with controls (*p<0.001). After receiving AT- or BM-MSCs, animals gradually regained weight, in contrast to vehicle-treated animals (**p<0.046; ***p<0.004) (B). Colonoscopic imaging was obtained after colitis induction at day 4, and after MSCs administration at day 11. In control experiments, colonoscopy was performed following intra-rectal saline enemas, and intraperitoneal administration of vehicle in TNBS-induced animals (C). Differences before and after treatment were evaluated with the Wilcoxon matched-pair signed rank test. Values are mean±S.E.M. of 10 animals/group.

In order to assess the severity of experimental colitis survival rates and changes in body weight were recorded. In regard to survival rates, deaths were observed in rats with TNBS-induced colitis, but none occurred in the AT-MSCs-treated animals. No statistical significance was found comparing survival rates among the different treatment groups (p = 0.225) (Figure 2A). As expected, body weight significantly decreased in TNBS-colitic animals treated with vehicle (cells solvent), compared with control rats (p<0.001). At days 7 and 11, weight loss in TNBS-induced animals significantly decreased following the treatment with AT-MSCs (p<0.03), and BM-MSCs (p<0.04), respectively, compared to vehicle-treated rats. In fact, MSCs-treated animals regained weight after day 4, in a slope comparable to the one observed for the control group (Figure 2B).

Colonoscopy was utilized for assessing the severity of colitis and for the detailed follow-up of mucosal changes after treatment. At day 4, animals were submitted to colonoscopy in order to confirm the presence and the severity of colitis individually. Dramatic changes were detected in all TNBS-induced animals, which displayed a fragile mucosa with intense hyperaemia, oedema, bleeding, deep and coalescent ulcerations, and necrosis. Animals were then assigned a group and treated thereafter with AT-MSCs, BM-MSCs, or vehicle. At day 11, animals were again submitted to colonoscopy. Among the colitic animals, colonoscopic evaluation significantly improved after treatment with AT-MSCs (p = 0.03) and BM-MSCs (p = 0.04), compared with vehicle-treated animals (Figure 2C and 2D).

### Intraperitoneal Inoculation of MSCs Induces Mucosal Healing in TNBS-Induced Colitis

**Figure 3:**
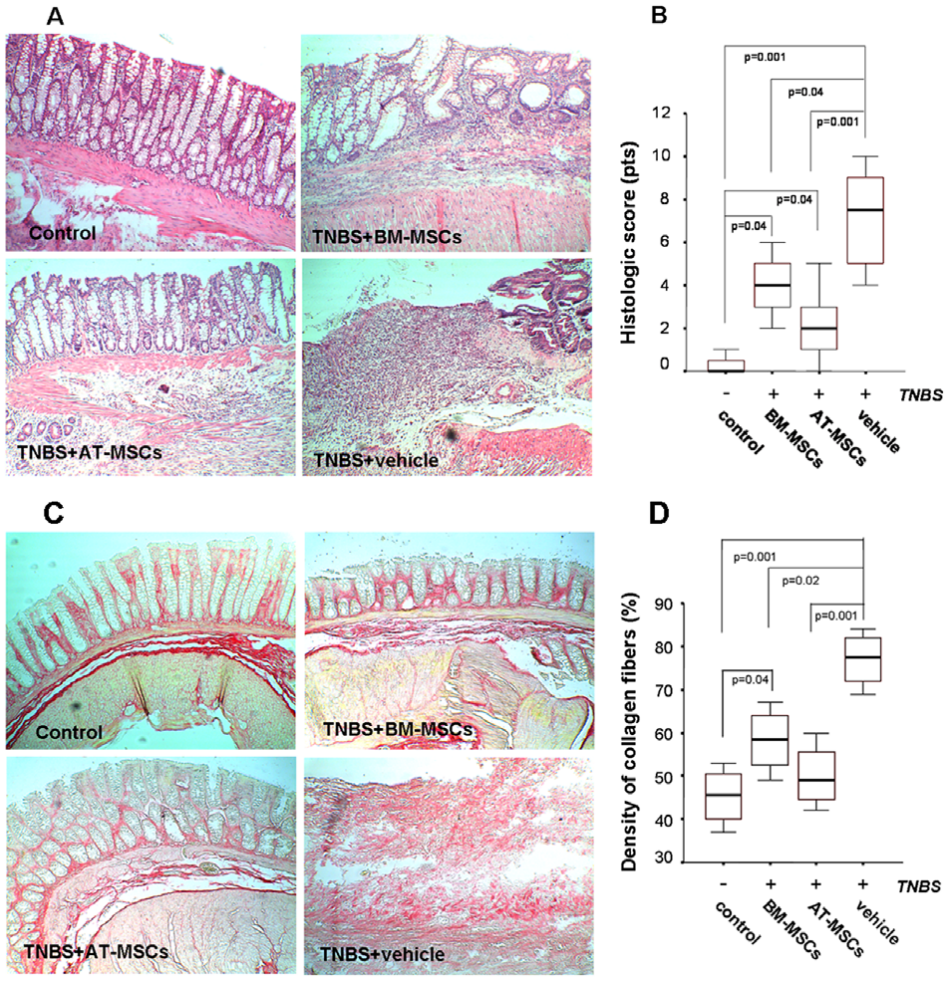
Effect of MSCs on histological parameters and collagen deposition in the colon. Paraffin sections were stained with HE (A), and phosphomolibidic acid-picrosirius red dye (C) (original magnification ×100). AT- or BM-MSC were administered intraperitoneally at day 4. In control experiments, histological evaluation was performed following intraperitoneal administration of vehicle, and intra-rectal saline enemas. Colonic samples were scored according to histological parameters described in materials and methods. Density of collagen fibres was determined by a computerized image analysis system. Horizontal bars represent medians, boxes represent the 25th and 75th percentiles, and vertical bars represent ranges, of 10 animals/group. Differences were analyzed using ANOVA on ranks with Dunnett's test (B, D).

Morphological changes of HE-stained intestinal sections were predominantly seen in the distal colon of TNBS-induced animals. TNBS-colitis was characterized by an intense cellular infiltration, predominantly of mononuclear cells, compatible with a Th1-mediated immune response. Analysis of intestinal sections revealed an increase in the microscopic damage score in TNBS-treated animals, compared with controls. Administration of MSCs significantly reduced the inflammatory scores in TNBS-treated animals (Figure 3A and 3B).

Collagen deposition in the colonic tissue was assessed by staining fibres with phosphomolibidic acid picro-sirius red dye. Collagen fibres were clearly disorganized in the injured colon, being diffusely distributed throughout the colon wall. Computer-assisted quantitative analysis of tissue sections showed that the density of collagen fibres was higher in the injured colon of TNBS-induced rats, and the injection of MSCs was able to significantly decrease collagen deposition within the colon, at levels comparable to the control group (Figure 3C and 3D).

**Figure 4:**
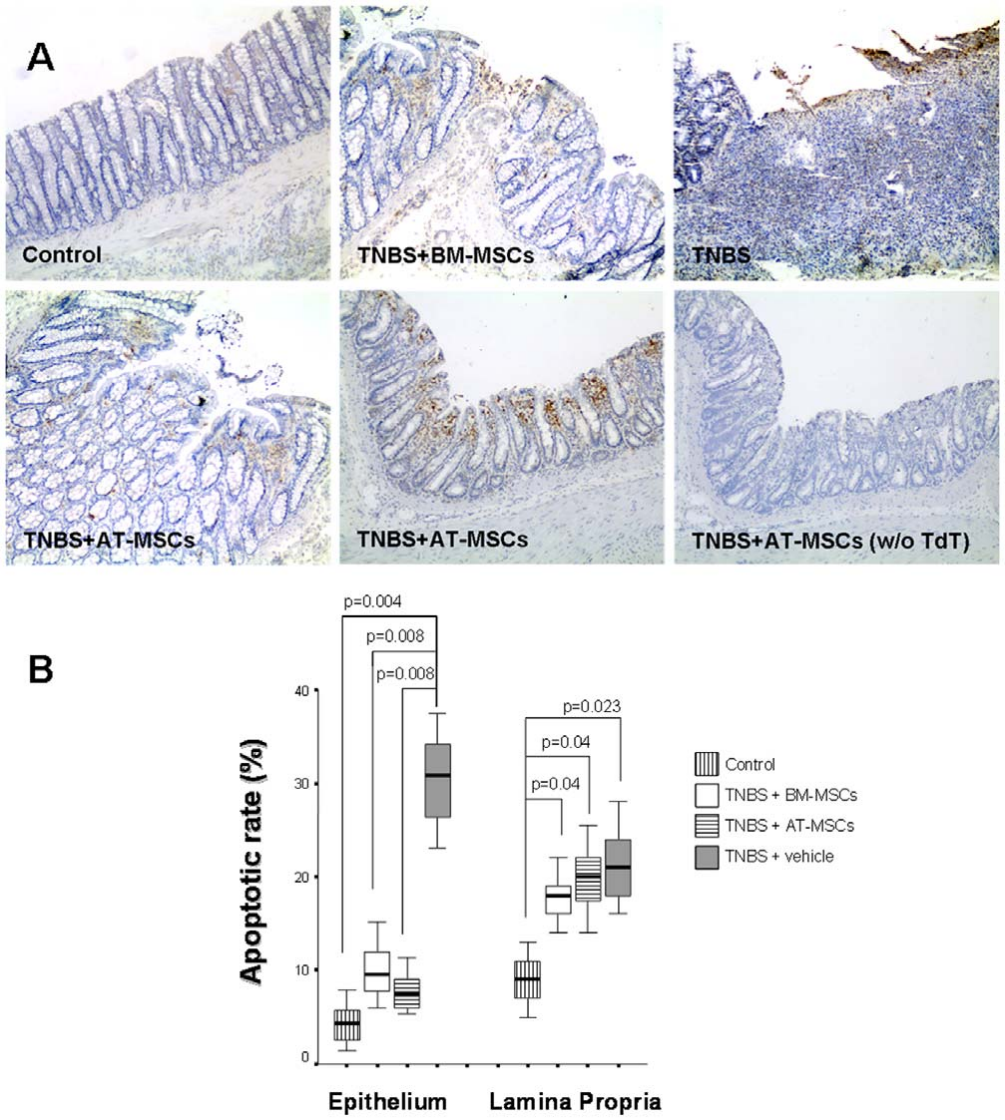
Effect of MSCs on apoptotic rates within the colon. Apoptotic cells were detected by the TUNEL assay. Photomicrographs of the colon show representative samples of a control, and from TNBS-induced colitic animals treated with BM- and AT-MSCs, or vehicle, and an internal negative control without TdT enzyme (original magnification ×100) (A). Percentages of apoptotic cells in the colonic epithelium and in the lamina propria were analyzed in at least 10 different areas per tissue section. Epithelial values of control and MSCs-treated animals were lower compared to vehicle-treated colitis (p<0.008). Lamina propria values of MSCs- or vehicle-treated animals were higher compared with the control group (p<0.04). Horizontal bars represent medians, boxes represent the 25th and 75th percentiles, and vertical bars represent ranges of 10 animals/group. Differences were analyzed using ANOVA on ranks with Dunnett's test (B).

In this TNBS-colitis model, a significant reduction in apoptotic cells was observed in the epithelium of MSCs-treated colitic animals compared to vehicle-treated TNBS-induced animals (p≤0.004). In the lamina propria compartment rates of TUNEL-positive mononuclear cells were significantly higher in animals treated with vehicle, BM-MSCs, and AT-MSCs compared to controls (p≤0.04). On the other hand, apoptotic rates in the lamina propria were not significantly different comparing MSCs- to vehicle-treated colitic animals (Figure 4A and 4B).

### Intraperitoneal Inoculation of MSCs Reduces Inflammatory Responses in TNBS-Induced Colitis

**Figure 5:**
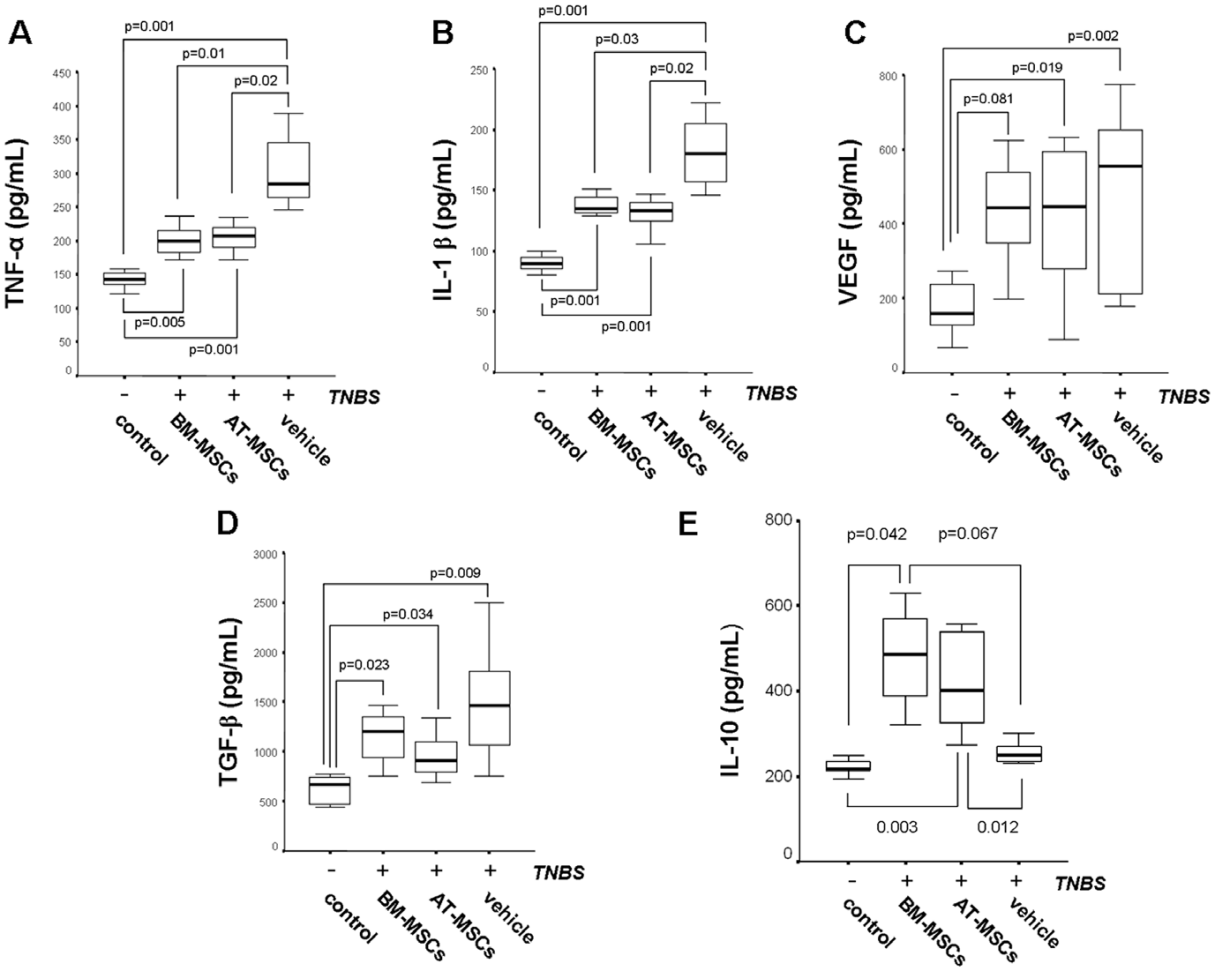
Effect of MSCs on cytokine production in the inflamed colon. Colonic explants were cultured for 24 h at 37°C. After centrifugation, supernatants were used for measurement of the concentration of cytokines by an ELISA method for rat TNF-α (A), IL-1β (B), VEGF (C), TGF-β (D), and IL-10 (E), as described in Supplementary Materials. AT-, BM-MSC, and vehicle were administered to colitic animals, while control group did not receive any treatment. Values are expressed as picogram of cytokine/mL of culture supernatant, and normalized by protein contents. Horizontal bars represent medians, boxes represent the 25th and 75th percentiles, and vertical bars represent ranges of 10 animals/group. Differences were evaluated with the one-way ANOVA on ranks with Dunnett's test.

Next, we investigated whether the administration of MSCs could affect the production of inflammatory mediators mechanistically involved in intestinal inflammation. TNBS treatment induced a ∼2-fold increase in TNF-α levels in the colon, which reduced significantly after the intraperitoneal administration of MSCs, regardless of its source (p≤0.02) (Figure 5A). Similarly, levels of IL-1β significantly reduced in inflamed colon after MSCs administration (p≤0.03) (Figure 5B). No significant difference was observed in the effect of AT-MSCs compared with BM-MSCs.

Angiogenesis has been regarded as an important element in IBD pathogenesis, and angiogenesis blockade was shown to attenuate inflammation [17]. Therefore, we sought to determine whether treatment with MSCs would affect VEGF production in the inflamed colon of the model. TNBS induced a 3.5-fold increase in VEGF production. Administration of MSCs irrespective of the origin (AT- or BM-derived) did not reduce significantly VEGF production to levels observed in control animals (Figure 5C).

TGF-β is a multifunctional cytokine capable of modulating epithelial cell restoration during active inflammation, and also of remodelling the extra cellular matrix following intestinal injury [18]. Here, we show that TNBS induced a ∼3-fold increase in TGF-β production, but levels did not reduce significantly after MSCs-treatment, independent of being AT- or BM-derived (Figure 5D).

IL-10 is a regulatory cytokine known to act as anti-inflammatory molecule, critical for tissue homeostasis [19]. In this study, we observed a significant increase in IL-10 levels after TNBS-induced animals have been treated with MSCs (Figure 5E).

### Trafficking and Distribution of MSCs *in vivo*

**Figure 6:**
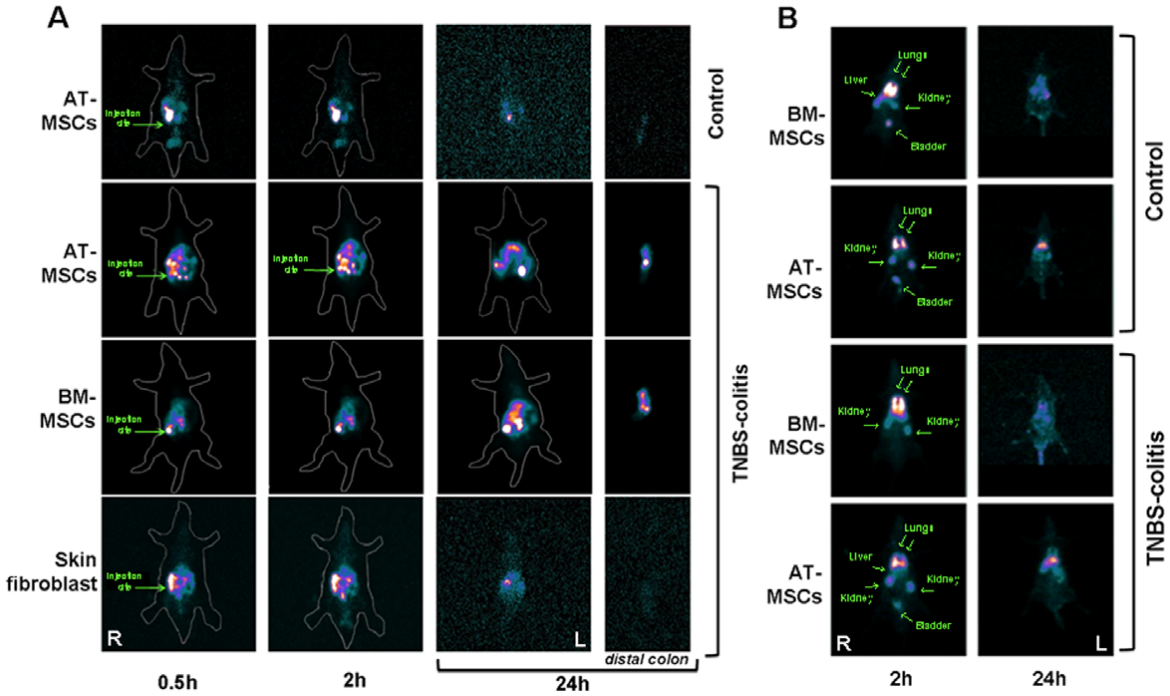
Migration of intraperitoneally and intravenously administered MSCs in experimental animals. Tec-99m-labeled MSCs were administered to animals and followed for 24 hours using a gamma-camera. Tec-99m-labeled MSCs were injected intraperitoneally in the right lower abdominal quadrant, and migrated towards the inflamed colon on the contra-lateral left lower quadrant, but not to the non-inflamed colon of controls. Skin fibroblasts used as additional controls did not migrate towards the inflamed colon (A). Tec-99m-labeled MSCs injected through the jugular vein accumulated first into the lungs and then gradually migrated towards liver, spleen, kidneys, and bladder. After 24 hours, labelled cells were barely detectable (B). Images are representative of 3 independent experiments. All animals are lying on their backs. Right (R); left (L).

Tc-99m labelled MSCs that were intraperitoneally administered into the right lower quadrant, moved within the first 2 h towards the opposite side of the abdomen of TNBS-induced animals, but not in controls. Twenty-four hours later, labelled cells accumulated in the surgically removed distal colon of TNBS-induced animals, but not in controls. Skin-derived fibroblasts used as additional controls did not migrate significantly to the inflamed colon (Figure 6A).

To evaluate possible differences in terms of the route of administration, Tc-99m-labeled MSCs were also intravenously administered to animals, through the right jugular vein.

As a result, labelled-MSCs rapidly accumulated in the lungs, and also distributed to the kidneys and liver in all animals within the first 2 hours. Twenty-four hours later, labelled cells were predominantly detected in the liver and kidneys, in addition to the spleen and bladder of all animals. No significant concentration of labelled cells was detected in the colon topography (Figure 6B).

**Figure 7:**
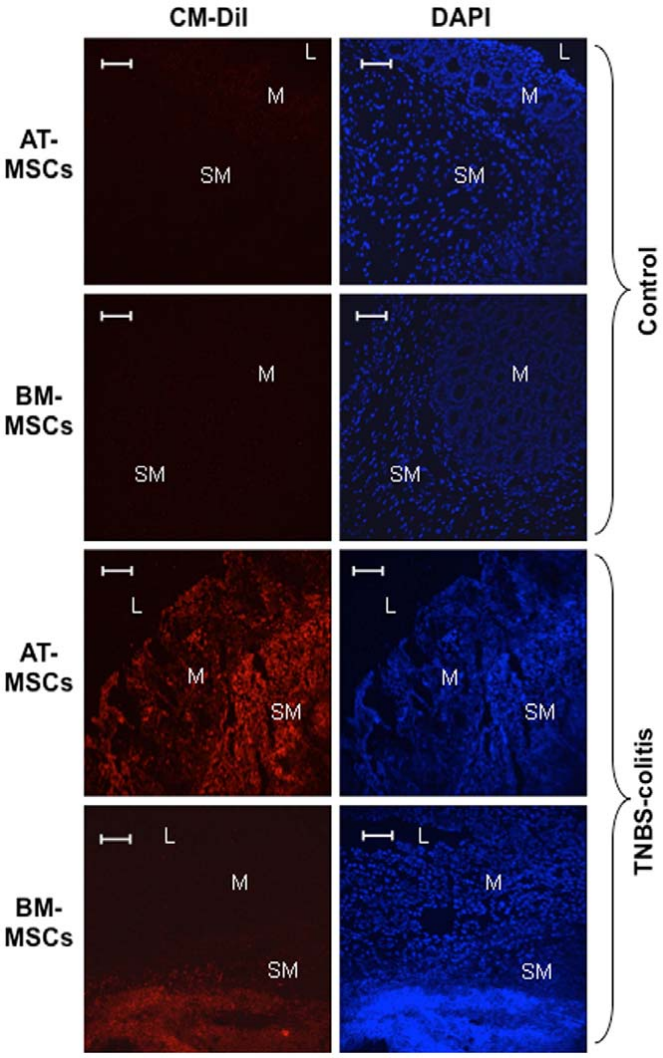
Distribution and engraftment of intraperitoneally administered MSCs in the colon. Cm-DiI-labelled MSCs (red) injected intraperitoneally in the right lower abdominal quadrant were tracked after 72 hours. After surgical removal of the distal colon, samples were cut in a cryostat, counterstained with DAPI (blue), and analyzed under confocal microscopy. Labelled cells migrated towards the inflamed colon on the contra-lateral left lower quadrant, but not to the non-inflamed colon of normal controls. Cm-DiI-labeled MSCs were mainly observed in the muscular layer and the submucosa (SM), but also in the lamina propria of the mucosa (M), predominantly in the bottom of crypts. AT-MSCs distributed throughout the colon wall, from the peritoneum to the epithelial layer, whereas BM-MSCs concentrated at the peritoneal surface, muscular layer and the submucosa. No labelled MSCs were observed in control animals. Intestinal lumen (L). Images are representative of 3 independent experiments. Length bars represent 100 µm.

To evaluate in more detail the tissue distribution of intraperitoneal injected MSCs which migrated towards the inflamed colon, we stained MSCs with a lipophilic fluorochrome, an indocarbocyanine (Cm-DiI). Hence, Cm-DiI is well retained and provides a greater and long-lasting stability and staining intensity compared to most other fluorochromes or radioisotopes, including Tc-99m [20]. The orange-red Cm-DiI-labeled MSCs were mainly observed in the muscular layer and the submucosa, but also in the lamina propria, predominantly in the bottom of crypts. AT-MSCs consistently distributed throughout the colon wall, from the peritoneum to the epithelial layer. On the other hand, BM-MSCs characteristically concentrated at the peritoneal surface, muscular layer and the submucosa. However, in control animals and in the non-inflamed tissue such as the proximal colon, no labelled MSCs were observed (Figure 7).

## Discussion

Currently, approved therapies for IBD have limited efficacy and side effects constitute a major concern from aminosalicylates and corticosteroids, to the new biological agents [3]. Although the pharmacological agents are still the mainstay of IBD treatment, safety issues and elevated costs of prolonged therapy encourage the search for alternative approaches. Here, we tested the therapeutic effect of non-myeloablative allogeneic MSCs transplantation in TNBS-induced colitis, a murine experimental model of CD. Of note, we compared different routes of administration and tested the functionality and potential beneficial effects of cryopreserved MSCs in experimental colitis. In respect of efficacy, both AT- and BM-MSCs consistently stabilized the animals' weight, while lowering the colonoscopic and histologic scores in the experimental model compared with colitic animals, after one week of treatment. In particular, we demonstrated that treatment with MSCs reduces the amount of collagen deposition, the rate of epithelial apoptosis, and the local production of proinflammatory cytokines. In addition, we delineated for the first time MSCs homing to inflammatory sites and the route-dependent differential distribution and engraftment of MSCs within the colonic tissue.

Similar to previous reports, in this study we have isolated MSCs from the subcutaneous adipose tissue and the bone marrow of Wistar rats, exhibiting several characteristics such as multipotent differentiation into different cell lineages, and the expression of mesenchymal cell surface markers [21]. In particular, the high rates of population doubling easily expanded ex vivo and the preserved functional properties after thawing, would enable us to use them in several clinical conditions suitable for cell-based therapy.

A potential caveat to the effective implementation of MSC-based therapy is the current inability to direct these cells to tissues of interest. In this study, we focused on the cell trafficking and tissue localization of exogenous MSCs delivered through different routes of administration. Similar to a previous study [22], we also demonstrated that intravenously administered MSCs rapidly accumulate into the lungs or within the liver and other organs, with no evidence of homing to the colon. In contrast, others have shown that bone marrow derived MSCs differentiate into sub-epithelial myofibroblasts in mice with TNBS-induced colitis following intravenous administration of cells [23], [24]. In another study with murine dextran sulphate sodium (DSS)-induced colitis, exogenous CD34-negative stem cells also migrated towards the inflamed colon and differentiated into endothelial cells following intravenous administration [25]. Discrepancies regarding homing experiments may result from different details in each protocol, including the number of cells and doses administered, the confluence of MSCs prior to infusion [26], the passage number, as MSCs have been shown to gain or loose surface receptors during culture [27], [28], and also the site of isolation, the properties of the media, and the route or site of injection. Because systemically administered MSCs did not reach the inflamed colon and had no beneficial effect in our TNBS-colitis model, we sought to investigate whether intraperitoneal administration would facilitate the homing and potential anti-inflammatory actions of MSCs with exactly the same characteristics. In rats with TNBS-induced colitis, submucosally injected Y-chromosome-positive AT-MSCs traced with FISH were detected under the basal membrane, in the muscle layer, and in adipose tissue, compatible with fibroblast-like or smooth muscle cell by immunohistochemistry [12]. Although even not injecting the cells in such a close proximity with tissue injury, we could still clearly demonstrate MSCs migration and infiltration into the inflamed colon, predominantly in the muscular layer and the submucosa, but also in the lamina propria and the subepithelial space of the mucosa. This is in contrast with a previous report of BM-MSCs being detected in the epithelium of the stomach and intestine [29], and the thought that MSCs in bone marrow would possibly be more primitive than AT-MSCs. Actually, our results support the concept that AT-MSCs have at least the same potential to migrate and engraft in the inflamed tissue, and probably to differentiate into various cell lineages in vivo as well as BM-MSCs. Furthermore, this is the first report to show in detail the path of intraperitoneal injected MSCs, migrating to inflamed sites, accumulating at the peritoneal lining of the inflamed colon, and passing through the whole intestinal wall towards the luminal side.

In respect of the anti-inflammatory actions of exogenous MSCs, downregulation of the Th1-driven inflammatory responses has been previously reported following treatment with human AT-MSCs of DSS-colitis [14], and TNBS-colitis [13]. In fact, proinflammatory cytokines of the Th1-type of immune response such as TNF-α and IL-1β are known to be present in high levels in CD [2] and in TNBS-colitis [16]. TNF-α is regarded as a crucial molecule for the development of the inflammatory process of CD, and the beneficial actions of anti-TNF-α agents reinforces the role of TNF-α in the pathogenesis as well as a therapeutic target in CD [30]. IL-1β is another proinflammatory cytokine of the Th1-type of immune response also known to be involved in the immediate innate immune responses to peptidoglycan products [31], [32]. In the present study, levels of both TNF-α and IL-1β decrease in colitic animals treated with MSCs in accordance to the notion that MSCs possess immunomodulatory effects [8], [33]. Independently of being considered as stem cells per se or of questions regarding the ability to differentiate into specific cell lineages, the successful anti-inflammatory action of MSCs may not require their sustained engraftment in tissue. As it has been proposed before, it is likely that immunomodulatory properties of MSCs may be mediated in a paracrine fashion in vivo [34], [35].

Previous investigations on the ability of MSCs to modulate the immune system were based initially on the suppression of T-cell effector functions upon coculture with MSCs [8], [36]. In another approach focused on the interaction of MSCs with leukocytes suggested that MSCs can increase suppressor populations, such as Foxp3^+^ regulatory cells [37], with consequent increase in IL-10 production [38], [39]. These findings have motivated the utilization of MSCs in a number of preclinical and clinical diseases. In experimental colitis, the use of MSCs have demonstrated beneficial effect in down-regulating proinflammatory cytokines and increasing regulatory T-cell populations [13], [14]. In addition to actions on regulatory T-cells, in an experimental model of sepsis MSCs were shown to induce macrophage reprogramming into a regulatory profile, with the ability to produce IL-10 [40]. In agreement with this, we also showed an increase in IL-10 production following MSCs treatment.

Although the exact mechanisms underlying MSC-mediated suppression of lymphocyte proliferation remain basically unknown, it is possible that MSCs can accelerate apoptosis of active inflammatory cells. In the intestinal mucosa, apoptosis constitutes a critical mechanism exerting control over lamina propria mononuclear cells and regulating immune responses [41]. In this study, persistently elevated rates of apoptosis in the lamina propria despite the remarkable mucosal healing may indicate continued apoptosis induction as a possible immunosuppressive mechanism of MSCs transplantation. However, in the epithelial compartment, increased rates of apoptosis were shown to result in disruption of the epithelial barrier, overexposing the gut to luminal antigens and thus initiating the systemic inflammatory response in experimental models of IBD [42], [43]. In the present study, a dramatic reduction of epithelial cell apoptosis in TNBS-colitis was also observed following intraperitoneal injection of MSCs. These results are consistent with previous studies indicating that TNF-α and IFN-γ regulate intestinal epithelial cells proliferation and apoptosis [44], [45], and suggest that exogenous MSCs target directly or indirectly key effectors in colon inflammation.

Persistently high TGF-β levels could contribute for down-regulating the immune response, but could also enhance intestinal fibrosis in the TNBS-model. Here, we showed that MSCs-treatment of TNBS-colitis did not change the levels of TGF-β, but significantly reduced collagen deposition. A possible explanation for this effect could be attributed to the complex interaction among the different mediators present in the context of colitis. For example, TNF-α present in high levels in CD intestinal mucosa, stimulate the production of metalloproteinases and collagen by myofibroblasts and fibroblasts, contributing to matrix degradation and fibrosis [46]. Therefore, it is likely that the immunomodulatory actions of MSCs, including TNF-α suppression could actually play an effective role in preserving the extra cellular matrix of the TNBS-colitis model. In analogy, MSCs transplantation has been shown to limit fibrosis of myocardial infarction and dilated cardiomyopathy [47], which has been attributed to adrenomedullin, regarded as an antifibrotic factor secreted by MSCs [32].

The formation of new blood vessels is a critical step in wound healing process [48]. Previously, it has been demonstrated that BM-MSCs-treated wounds resulted in augmented capillary density, an effect promoted by conditioned medium, suggesting a paracrine effect of BM-MSCs, which also expressed high levels of VEGF and Angiopoetin-1 [49]. Recent studies provided evidence for the involvement of angiogenesis in the pathogenesis of IBD. The expression of the proangiogenic factor VEGF was shown to be increased in intestinal mucosa of patients with active CD [17]. Corroborating literature data, here we showed a marked increase of VEGF levels in the inflamed colon following TNBS exposure. Administration of MSCs did not reduce significantly the growth factor levels to that of control animals, suggesting that the anti-inflammatory actions are independent of VEGF, which in turn could promote and sustain angiogenesis in the model [50].

In conclusion, the successful treatment of experimental colitis with the intraperitoneal administration of cryopreserved allogeneic MSCs supports the idea of using these cells for treating intestinal inflammation. In particular, considering that cells share the same therapeutic properties, the use of AT-MSCs appears to be more convenient than BM-MSCs because of ease and safety of isolation by liposuction and the abundant tissue source of AT-MSCs. In addition, the rapid ex vivo expansion and the possibility of cryopreservation render these AT-MSCs a useful source for cell therapy, emerging as a potential new alternative of non-myeloabaltive cell-based therapy for human IBD.

## Materials and Methods

### Ethics Statement

The institutional animal care committee of the Federal University of Rio de Janeiro approved the care and use of animals, as well as procedures reported in this study (approval number # 66/08), in accordance with the guidelines of the International Care and Use Committee of the National Institutes of Health, and Guide for the Care and Use of Laboratory Animals (National Research Council-USA, 1996).

### Animals

Male Wistar rats (each weighing between 250 and 300 g) obtained from the Animal Care of the Laboratory of Experimental Surgery of the Federal University of Rio de Janeiro (Rio de Janeiro, RJ, Brazil) were maintained under specific pathogen-free conditions in a temperature-controlled room (24°C), on a 12-h/12-h light and dark cycle. Standard laboratory pelleted formula and tap water were provided *ad libitum*. Animals were housed in rack-mounted wire cages with 2 animals per cage.

### Mesenchymal Stromal Cells Isolation and Culture

Bone marrow cell suspensions were obtained by flushing marrow cavity of rats with Dulbecco's modified Eagle medium-low glucose (DMEM) (LGC Biotecnologia, São Paulo, SP, Brazil). Cells were plated in 25 cm^2^ culture flasks with DMEM supplemented with 15% Foetal Bovine Serum (Cultilab, Campinas, SP, Brazil) and antibiotics (100 U/mL of sodium-penicillin and 100 mg/mL of Streptomycin, both from Sigma-Aldrich, St. Louis, MO, USA). After 2 to 3 days in culture, the non-adherent cells were removed and adherent cells were maintained in culture until they reach 70% confluence. The monolayer was trypsinized and expanded. AT-MSCs were obtained as previously described [51]. Briefly, subcutaneous inguinal adipose tissue was removed, dissected from visible blood vessels, and enzimatically digested at 37°C in DMEM with 2 mg/mL of collagenase type II (Sigma-Aldrich, MO, USA). Cell suspensions were centrifuged, and the pellet was resuspended in DMEM with 20% FBS and antibiotics, and filtered in nylon mesh of 100 µm. Cells were then plated in 25 cm^2^ culture flasks and incubated overnight at 37°C with 5% CO_2_. The non-adherent cells were removed and the plastic-adherent cell population was maintained and expanded as described above. After the third passage MSCs were then stored in liquid nitrogen until being used in the experiments. For this purpose, cryopreserved cells were first thawed, washed twice and resuspended in phosphate buffered saline (PBS) 0.1% bovine serum albumin (BSA) (vehicle).

### Osteogenic Differentiation

Osteogenic differentiation was induced by adding to pre-confluent monolayers DMEM supplemented with 10% FBS, 50 µM ascorbic acid, 10 mM β-glycerophosphate, 10^−8^ M dexamethasone and antibiotics (all from Sigma-Aldrich Inc., St. Louis, MO, USA). Cells were maintained in osteoinductive medium containing dexamethasone for 1 week with alternate day media exchange. After this period, dexamethasone was removed and the cells were maintained as described above for additional 2 weeks with alternate day media exchange. Calcium deposition was revealed by Von Kossa staining [52].

### Adipogenic Differentiation

To induce adipogenic differentiation, cells were cultured for 2 weeks with DMEM supplemented with 10% FBS, 10^−8^ M dexamethasone, 100 µM indomethacin, (all from Sigma-Aldrich Inc., St. Louis, MO, USA) and 2.5 µM insulin (Biohulin®, Biobrás S.A., M. Claros, MG, Brazil). Cytoplasmic lipid deposits were stained with Oil Red O solution (0.5% in isopropanol) for 20 minutes at room temperature. Cultures were counterstained with Harris' haematoxylin.

### Flow Cytometry

Cell suspensions were washed with PBS with 3% FBS and 0.1% sodium azide (staining buffer) and incubated for 30 min at 4°C with fluorescein (FITC), R-phycoerythrin (R-PE), or Cychrome 5 (Cy5) conjugated monoclonal mouse anti-rat antibodies, CD11b (Mac-1, clone OX-42) from CaltagTM Lab (Invitrogen Life technologies, USA), CD34 (clone ICO115) from Santa Cruz Biotechnology Inc. (Santa Cruz, CA, USA), CD29 (clone Ha 2/5), CD45 (clone OX-1), and CD90 (clone OX-7), all from BD-Pharmingen (San Jose, CA, USA). Cells were washed with the staining buffer and acquisition was performed on a FACSCalibur using the CellQuest Pro software (BD Biosciences, San Jose, CA, USA).

### Skin Fibroblast Isolation and Culture

Skin fibroblasts were obtained by skin explants outgrowth. The skin was shaved and cleaned, and the subcutaneous tissue was removed. Skin fragments of 1 to 2 mm^2^ were plated in 2 ml of DMEM supplemented with 10% FBS and antibiotics. Cultures were incubated overnight upside down. After this period the flasks were carefully turned and fresh medium was added. Cultures were maintained and expanded as described above.

### Induction of Colitis

On day 0, rats were anesthetized subcutaneously with ketamine (35 mg/kg) and xylazine (5 mg/kg), and colitis was induced by intracolonic instillation of 1 ml of a solution containing 25 mg of 2,4,6-trinitrobenzene sulfonic acid (TNBS) (Sigma Chemical Co., St. Louis, MO, USA) in 30% ethanol (Merck, Darmstadt, Germany) using a 4-French catheter (8-cm long) inserted through the rectum, as previously described [53]. Thereafter, animals were allowed access to standard chow and water *ad libitum*.

### Experimental Design

After an acclimation period of one week, rats maintained under specific pathogen-free conditions were randomly assigned to one of four groups of 9–12 animals each. On day 0, colitis was induced by the TNBS/ethanol administration. Rats receiving intracolonic injection of phosphate buffered saline constituted the normal control group. During the whole experiment clinical manifestations such as diarrhoea, bleeding, and weight changes were recorded. On day 4, rats were anesthetized as described in the previous paragraph and submitted to colonoscopy. Animals with established colitis received AT-MSCs, BM-MSCs, or the cells' vehicle. Because the effects of 0.5 and 1×10^6^ cells were only modest in our preliminary experiments, we established 2×10^6^ cells as the number of cells to be used in all experiments thereafter. MSCs (2×10^6^ cells/0.3 mL) were administered in a single intraperitoneal injection on day 4. On day 11, a follow-up colonoscopy was performed, prior to tissue collection. For the surgical procedure, animals were anesthetized as previously described and submitted to a laparotomy under sterile technique. The distal colon was removed, opened longitudinally, rinsed with saline, and tissue samples were excised for histological assessment. A quick death procedure by cervical dislocation was uniformly performed in all animals on experimental day 11.

### Colonoscopic Assessment

On experimental days 4 and 11, animals were anesthetized and examined with a flexible fiberscope FB 120P with a diameter of 2.8 mm (Fujinon) assembled to a video-camera for recording images. Colon injury was scored by two independent observers using an adapted endoscopic index of colitis [54], [55]. The following parameters were analyzed and graded as 0 (absent) or 1 (present) each: changes of the vascular pattern; mucosal granularity; strictures; bleeding; and ulcers (total score ranging from 0–5).

### Histological Inflammatory Scores of the Colon

Specimens were fixed in 40 g/liter formaldehyde saline, embedded in paraffin, cut into 5-µm sections, stained with haematoxylin-eosin stain, and examined microscopically by two independent observers. The following histological parameters were studied: ulceration, hyperplasia, and inflammatory infiltrate. For both inflammatory infiltrate and hyperplasia, grading was considered: 3, severe; 2, moderate; 1, mild; 0, absent. For ulcers, grading was: 4, diffuse glandular disruption or extensive deep ulceration; 3, glandular disruption or focal deep ulceration; 2, diffuse superficial ulceration; 1, focal superficial ulceration; and 0, absent [56].

### Assessment of Collagen Deposition in the Colon

Specimens were fixed in 40 g/litre formaldehyde saline, embedded in paraffin, and cut into 5-µm sections. The phosphomolibidic acid picro-sirius red dye was used to stain collagen fibres in tissue [57]. At least 10 different areas per tissue section were analyzed under light microscopy connected to a computer-assisted image analyzer.

### Assessment of Apoptosis in the Colon

To determine apoptosis, fragmented DNA was stained by the terminal deoxynucleotidyltransferase (TdT)-mediated dUDP-biotin nick end labelling (TUNEL) assay, with the TACS TM TdT kit - in situ apoptosis detection kit (R&D Systems, Minneapolis, MN, USA). Paraffin sections were first deparaffinized, incubated with proteinase K solution for 15 minutes at room temperature, and then immersed in hydrogen peroxide to block endogenous peroxidase activity. After rinsing, slides were incubated with the TdT labelling buffer for 5 minutes. This step was followed by the incubation with the labelling reaction Mix containing TdT enzyme for 1 hour at 37°C. The biotinylated nucleotides incorporated to DNA fragments were detected using streptavidin horseradish peroxidase conjugate. A second section of each sample, incubated without TdT enzyme, constituted the negative controls. Positive controls were prepared by treating samples with TACS-nuclease. After being rinsed in PBS, all sections were developed with a solution containing hydrogen peroxide and diaminobenzidine. Preparations were lightly counterstained in Harris's haematoxylin, dehydrated, and mounted in Permount (Fisher Scientific, Pittsburgh, PA, USA). Morphologically preserved TUNEL-positive cells and apoptotic bodies were referred to as apoptotic cells and determined by using predefined measurements in the computer-assisted image analyser in conjunction with careful evaluation of morphologic criteria.

### Quantitative Assessment of Colon Sections

Quantitative analysis of tissue sections (under light microscopy) was carried out using a computer-assisted image analyser (Leica QWin Plus V 3.5.1, Leica Microsystems Ltd, Switzerland). The density of collagen fibres was defined by the area positively stained for collagen in relation to total intestinal tissue per millimetre- squared using an imaging analysis system at (×100 magnification). Percentages of apoptotic cells were defined by the number of immunoreactive cells in relation to total cells (immunoreactive and non-immunoreactive cells; ×400 magnification) in the lamina propria per millimetre squared (counted in at least 10 different areas), or in at least 500 epithelial cells in the crypts and in the surface epithelium of longitudinally sectioned colonic crypts. Two independent observers who were unaware of the experimental data examined all tissue sections and captured images.

### Organ Culture and Cytokine Measurements

Colonic mucosal explants were cultured in RPMI 1640 medium supplemented with 10% foetal calf serum (Gibco-Invitrogen, Carlsbad, CA, USA), 2 mM L-glutamine, 50 µM 2-mercaptoethanol, 10 mM HEPES, penicillin (100 killiunits/litre) and streptomycin (100 mg/litre) (all from Sigma Chemical Co., St. Louis, MO, USA) for 24 h at 37°C in a 5% CO_2_ humidified incubator. Samples were centrifuged and the supernatants used for measurement of the concentration of cytokines TNF-α, IL-1β (both from Peprotech Inc., Rocky Hill, NJ, USA), VEGF and TGF-β (R&D Systems, MN, USA), and IL-10 (Invitrogen, Camarillo, CA, USA) by commercial sensitive enzyme-linked immunosorbent assays (ELISA) method. The total protein content of the biopsy specimens was estimated by the Pierce® BCA protein assay kit (Thermo Scientific, Rockford, IL, USA), and used for normalizing the results. The minimum detectable concentration of rat TNF-α, IL-1β, VEGF, TGF-β, and IL-10 was less than 5.0 pg/mL.

### MSCs Labelling and Imaging

For cell migration studies in vivo, AT-MSCs, BM-MSCs, and skin fibroblasts were labelled with Tc-99m or stained with CM-DiI before injection on day 4, and traced for up to 72 hours thereafter.

Briefly, for each animal 2×10^6^ cells (0.3 mL) were labelled with Tc-99m based on previously published protocols [58], [59] under sterile conditions. Initially, 500 µl of SnCl_2_ solution was added to cell suspension in 0.9% NaCl, and the mixture was incubated at room temperature for 10 min. Then, 5 mCi Tc-99m (1 mL) was added and the incubation continued for another 10 min. After centrifugation (500×g for 5 min), the supernatant was removed and cells were washed again and resuspended in saline solution. Viability of the labelled cells was assessed by the trypan blue exclusion test and estimated to be greater than 93% in all cases. Labelling efficiency was calculated by the activity in the pellet divided by the sum of the radioactivity in the pellet plus supernatant, and was estimated to be greater than 90% in all cases. Tc-99m-labeled cells were then injected intraperitoneally or intravenously through the jugular vein. Labelled cells were followed-up with scintigraphic images taken at 30 min, 2 and 24 h after injection at a dual head gamma-camera equipped with two high-resolution collimators (Millennium MG General Electric Medical Systems, Milwaukee, WI). Animals were sacrificed under anaesthesia and bowels were scanned and counted at a Perkin-Elmer Packard Cobra II Auto Gamma-counter. Representative images were obtained and analyzed.

In another set of experiments, MSCs were stained with the fluorescent Cell Tracker™ CM-DiI (indocarbocyanine) (Invitrogen, Carlsbad, CA). Briefly, monolayers were incubated with 2 µg/mL of CM-DiI in DMEM with 10% FBS at 37°C for 5 minutes followed by 15 minutes at 4°C. Cells were then washed with PBS and trypsinized. For assessing tissue distribution of CM-DiI-labeled MSCs, 2×10^6^ cells were injected into the peritoneal cavity on day 4. Skin fibroblasts used as additional controls did not migrate into the inflamed colon (data not shown). Seventy-two hours after cells administration, the left colon was surgically removed and tissue samples were embedded in resin and snap-frozen in isopentane in a liquid nitrogen bath. Samples were stored at −80°C until processing, and then cut into 6-µm section in a cryostat maintained at −20°C. Tissue sections were air-dried, fixed for 5 min in 1% paraformaldehyde, and mounted in an antifading medium containing DAPI. Slides were analyzed with a Leica TCS-SP5 AOBS confocal laser scanning microscope for capturing representative images of each sample.

### Statistical Analysis

Statistical analyses were performed using the statistical software SPSS for Windows (Version 10.1, SPSS Inc., 1989–1999, USA). Statistical differences among the experimental groups were evaluated with the one-way ANOVA on ranks test in which pair wise multiple comparisons were carried out using the Dunnett's T3 test. Colonoscopic scores and body weight changes before and after treatment were compared by the Wilcoxon matched-pair signed rank test. Survival was analyzed by the Kaplan–Meier log-rank test. The level of significance was set at p<0.05.
